# Quadri-stability of a spatially ambiguous auditory illusion

**DOI:** 10.3389/fnhum.2014.01060

**Published:** 2015-01-15

**Authors:** Constance M. Bainbridge, Wilma A. Bainbridge, Aude Oliva

**Affiliations:** ^1^Computer Science and Artificial Intelligence Laboratory, Massachusetts Institute of TechnologyCambridge, MA, USA; ^2^Department of Brain and Cognitive Sciences, Massachusetts Institute of TechnologyCambridge, MA, USA

**Keywords:** quadri-stable illusion, auditory spatial processing, sound localization, perceptual biases, front-back confusions

## Abstract

In addition to vision, audition plays an important role in sound localization in our world. One way we estimate the motion of an auditory object moving towards or away from us is from changes in volume intensity. However, the human auditory system has unequally distributed spatial resolution, including difficulty distinguishing sounds in front vs. behind the listener. Here, we introduce a novel quadri-stable illusion, the *Transverse-and-Bounce Auditory Illusion*, which combines front-back confusion with changes in volume levels of a nonspatial sound to create ambiguous percepts of an object approaching and withdrawing from the listener. The sound can be perceived as traveling transversely from front to back or back to front, or “bouncing” to remain exclusively in front of or behind the observer. Here we demonstrate how human listeners experience this illusory phenomenon by comparing ambiguous and unambiguous stimuli for each of the four possible motion percepts. When asked to rate their confidence in perceiving each sound’s motion, participants reported equal confidence for the illusory and unambiguous stimuli. Participants perceived all four illusory motion percepts, and could not distinguish the illusion from the unambiguous stimuli. These results show that this illusion is effectively quadri-stable. In a second experiment, the illusory stimulus was looped continuously in headphones while participants identified its perceived path of motion to test properties of perceptual switching, locking, and biases. Participants were biased towards perceiving transverse compared to bouncing paths, and they became perceptually locked into alternating between front-to-back and back-to-front percepts, perhaps reflecting how auditory objects commonly move in the real world. This multi-stable auditory illusion opens opportunities for studying the perceptual, cognitive, and neural representation of objects in motion, as well as exploring multimodal perceptual awareness.

## Introduction

Illusions are a delight to our playful minds, and artists, magicians, and scientists have long been searching for ways to create multiple meanings out of a single picture, sound, video or physical object. In particular, bi-stable and multi-stable perceptual illusions (e.g., *Rubin Vase*, *Necker Cube*, *Ames Window*, the *Spinning Dancer*) have revealed how versatile and flexible human perception can be: when multiple interpretations of an external stimulus are possible, the observer might spontaneously switch between two representations of the same physical stimulus (Sterzer et al., 2009). While most of these documented illusions are visual, multi-stable auditory illusions exist as well. For instance, in the *Shepard Tone* illusion (Shepard, 1964; Deutsch, 1992), the perceived pitch of a sound paradoxically rises or drops continuously.

A foremost advantage of audition compared to vision is that it represents a three-dimensional sphere surrounding the listener, rather than being restricted to the frontal hemisphere. This allows for the perception of auditory sound sources not only in the front or to the sides, but also behind the observer. For instance, the Virtual Barber Shop (1996) illustrates a virtual auditory space, where the sounds of a haircut are modulated binaurally to create a surrounding percept.

In auditory space, there exist zones of systematic ambiguity termed “cones of confusion” (Begault, 1994), where sounds create identical interaural time differences (ITD) and interaural level differences (ILD, see Section Materials and Methods). Within these cones of confusion, observers will often mistake a frontally located sound as originating behind the observer, or vice versa, due to equal ITDs and ILDs. While in the real world, head movements or additional spectral cues can resolve the multi-stable percepts (Wightman and Kistler, 1999; Brimijoin and Akeroyd, 2012), without these the confusion is common (Wenzel et al., 1993). In the near field, within distances of about one to two meters, ILDs provide unique position information distinct from that provided by ITDs, allowing a listener to constrain the range of sound sources that have identical ITDs. Thus, the cones of confusion are truncated to so-called “tori of confusion” (Shinn-Cunningham et al., 2000). The potential for confusion along the median plane, however, remains preserved as ITDs and ILDs are minimal.

**Figure 1:**
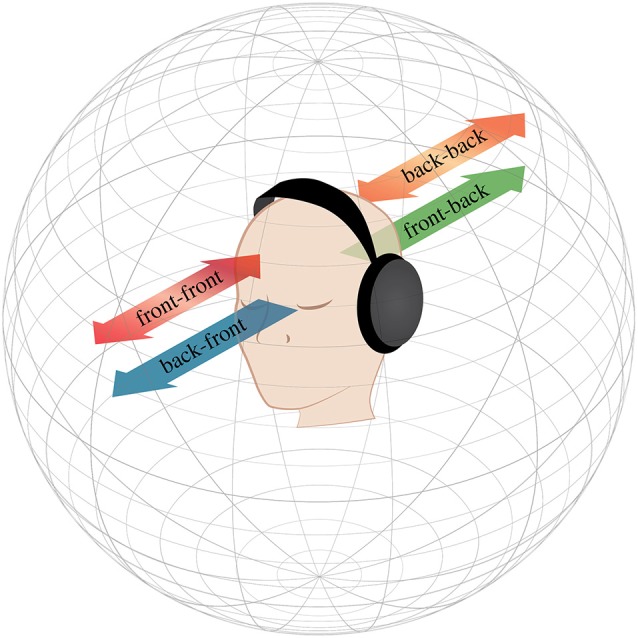
**The transverse-and-bounce illusion uses front-back confusion and volume changes to make a single sound stimulus that can be perceived as traveling in four different possible trajectories**. As the volume increases, the sound is perceived as approaching, and as it decreases, the sound recedes from the listener. As a result, the illusion can be perceived as traveling from: (1) front, through the listener, to the back, (2) back, through the listener, to the front, (3) front, to the listener, and returning to the front; or (4) back, to the listener, and returning to the back. These four percepts can be conceptualized as “transverse” percepts (front to back and back to front, in blue/green) that pass through the listener, and “bounce” percepts (front to front and back to back, in red/orange) that bounce off of the listener. This illusion can be played in speakers (Experiment 1) as well as headphones (Experiment 2).

Can this confusion also occur with the localization of moving sounds? In the visual world, objects approaching and receding from the observer can create ambiguous motion or be used to form ambiguous percepts of the object (approaching vs. receding ambiguity, Lewis and McBeath, 2004; the looming effect, Schiff et al., 1962; Neuhoff, 2001; hybrid images, Brady and Oliva, 2012). A similar phenomenon occurs in the auditory domain: changes in volume level can be interpreted as changes in an object’s distance, causing a sound to be interpreted as drawing nearer or farther away from the observer (the Growing-Louder Effect, Rosenblum et al., 1987; Middlebrooks and Green, 1991; Reinhardt-Rutland and Ehrenstein, 1996), which has been the basis for a number of perceptual illusions that affect localization (Small, 1977; Reinhardt-Rutland, 1996, 2004; Reinhardt-Rutland and Ehrenstein, 1996; Malinina and Andreeva, 2013). Coupled with the mechanism of the front-back confusion, a change in volume may influence people’s percepts of the trajectory of an imaginary object moving through space. This is exactly what we did here: we created an ambiguous auditory stimulus—what we call the *Transverse-and-Bounce Auditory Illusion—*lasting a few seconds, which produces the illusion of an object moving towards or away from the observer, along the front/back axis, by increasing and decreasing the signal amplitude (see also Vartanyan and Andreeva, 2007). Importantly, this produces a percept that is quadri-stable in nature, where the listener may perceive the same input sound as approaching from or receding towards the front or back. As illustrated in Figure 1, the listener may interpret the same sound as traveling in a transverse state from front-to-back or back-to-front, or as a sound bouncing exclusively in the front or exclusively in the back. This provides an auditory comparison to Metzger’s bi-stable moving balls, which appear to either cross trajectories or bounce off of each other (Metzger, 1934).

We conducted two human behavioral experiments that analyze the properties and potential biases in the quadri-stability of this illusion. In the first experiment, participants made several comparisons between the illusion and unambiguous stimuli of the illusion’s four possible percepts, using a free-field localization paradigm. We found that participants were unable to distinguish the illusion from its unambiguous counterparts (of sounds panning between speakers), and that they were able to equally perceive all four possible motion percepts of the illusion. In the second experiment, we examined perceptual biases for the illusion when it is looped continuously in headphones. We found that participants have a significant bias for perceiving a transverse motion vs. one that bounces off the listener, likely reflecting how objects move in the real world. Our results demonstrate that the Transverse-and-Bounce Auditory Illusion is a robust way to affect the spatial localization of auditory objects. Such an illusion has several merits, including the study of object perception outside of the visual field (i.e., behind the listener), and the study of top-down vs. bottom-up processing influences of auditory sound localization.

## General principle of the quadri-stable percept

The quadri-stable auditory percept is based on the combination of qualities from two phenomena: the front-back confusion and the Growing-Louder Effect (Reinhardt-Rutland and Ehrenstein, 1996). Volume and time cues—specifically, the ITD and ILD—allow one to determine the azimuth (i.e., horizontal angle) of a sound in space (Rayleigh and Strutt, 1907). To localize pitch and distance, the auditory system uses complex cues such as spectral qualities of the sound (e.g., the Doppler effect), spectral filtering due to the shape of the ear (Kondo et al., 2012), and the direct-to-reverberant energy ratio of the sound (Chowning, 1971). However, despite the complexities of the human auditory system, there are also sounds that are ambiguous in terms of their localization, resulting in front-back confusion (Stevens and Newman, 1936). These are sounds that have identical ITDs and ILDs but come from opposite sides of the head (back vs. front). For example, a sound located 5° right of the median plane may be mislocalized as being at 175°, as both azimuths have the same path length and level differences. This confusion can be resolved by making head movements to localize the sound (Kondo et al., 2012). In the previous example, turning one’s head 5° to the right would move the apparent position of the sound to 0° or 170°, two azimuths with readily distinguishable ITDs and ILDs. However, moving the sound contrarily to head movements can produce front-back confusion unimpeded by head movements (Brimijoin and Akeroyd, 2012). Additionally, an increase and decrease in sound intensity creates the perception of sound approach and recession (Reinhardt-Rutland and Ehrenstein, 1996). Combining these gradual sound intensity changes with the concept of front-back confusion allows for the creation of a sound that can be ambiguously perceived as approaching or withdrawing from the observer from the front or the back.

## Experiment 1—testing the illusion in a customized environment

**Figure 2:**
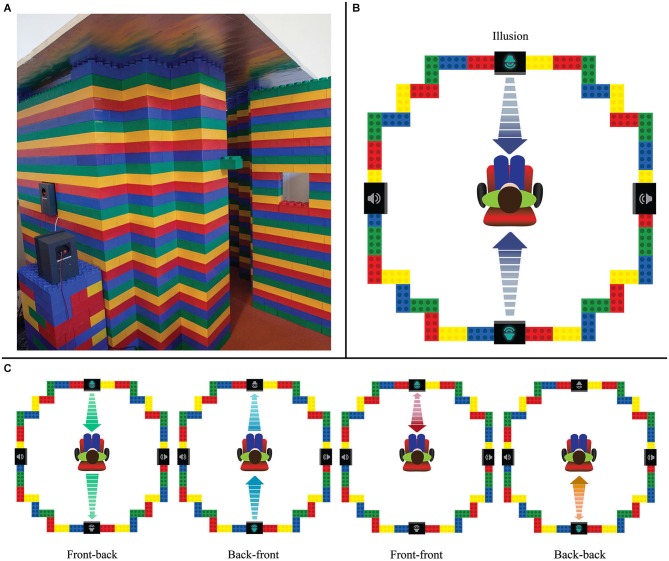
**(A)** The custom chamber for Experiment 1 is square and symmetrical; it avoids visual directional cues and keeps each speaker at an equal height and distance from the participant. Here, its door is open, but when the participant is inside, the door closes and the last speaker (bottom left) slides in, to make the room identical along all four walls. **(B)** The illusion is made by an increase and decrease in volume in both front and back speakers. Front-back confusion makes the sound’s location ambiguous, resulting in four different percepts. The arrows here indicate that the volume of the sound increases simultaneously in both front and back speakers, although it is perceived to approach the listener ambiguously from either the front or back. When the volume decreases (again, simultaneously in both speakers), the sound is perceived to withdraw away from the listener, but remains ambiguous as to whether it moves towards the front or back. **(C)** The four unambiguous stimuli match the four different possible percepts of the illusion: (from left to right) traveling in a single direction from either front-to-back or back-to-front, or bouncing off the listener exclusively in front or in back. For example, the front-back comparison stimulus begins only in the front speaker, with a peak volume at the listener, and ends only in the back.

In a first experiment, participants completed a series of tasks designed to compare perceptions of the illusion with the perception of unambiguous stimuli in the real world. To do so, we built a customized environment, designed to be symmetrical around the participant, both visually and in terms of auditory qualities (e.g., reverberation). As illustrated in Figure 2, we built a customized chamber from large plastic *Jumbo blocks* (similar to *Legos*), with four speakers positioned on the cardinal axes, at the height of the listener’s head, when sitting inside the chamber. Note that the critical point here was not to block out all reverberations of the sound (as in an anechoic chamber), but to distribute any potential echoes in an isotropic manner, so that any reverberation remained the same everywhere. Additionally, a key point was to test the illusion vs. stimuli moving in the real-world (rather than other artificially moving auditory stimuli). The jumbo blocks were ideal for such a chamber design, as they encapsulate sound within the chamber well, create a symmetrical visual environment, and can be easily reshaped to create new environments for future studies (see details in the Section Materials and Methods below).

Observers performed a series of tasks: first, without knowing the existence of an illusion, we verified participants could perceive both the illusory and unambiguous sounds as moving along a specified trajectory. Second, they heard illusory and unambiguous sounds and guessed their trajectories. Third, the illusion was revealed to them, and they tried to determine if sounds were “illusion” or “real” (i.e., unambiguous). A final control study was run with the participants listening to the illusion in their left and right ears, to ensure they could perform at ceiling at this now unambiguous task, and that any performance results found were intrinsic to the illusion and not the methods of the experiment.

### Materials and methods

#### Participants

Twenty-five (twelve female) participants between the ages of 18–35 were used in the study. All participants consented to the experiment following guidelines set by the MIT Institutional Review Board. A pure-tone audiometry (Wolfe et al., 2005) was used to prescreen participants to ensure that they were able to hear in the frequency and volume level of the stimulus, testing each ear independently. Although binaural hearing thresholds were not explicitly tested in a full spectrum audiometry, all participants self-reported normal hearing. Participants were compensated 20 h.

#### Testing apparatus

The testing location for the main experiment after the hearing test was a custom square chamber equipped with speakers in each of the cardinal axes (Figure 2A). The chamber was designed to be identical (and thus symmetrical) along these axes, with similar visual and auditory (e.g., reverberation) characteristics, in order to avoid external influences on the interpretation of the illusion or unambiguous comparison stimuli. The chamber was dimly lit to prevent overdependence on vision, and participants were seated in a chair measured to be in the middle of the room. Once the participant was inside, the door of the chamber was slid shut and a speaker was placed in it, to create a wall that looked visually identical to the opposite side. However, there was the possibility that the edges of the door could slightly alter the perception of sounds in its direction. To control for this possibility, half (*N* = 13) of the participants performed the experiment while facing away from the chamber door, while the remainder performed the experiment facing towards the chamber door.

Testing was performed using MATLAB and Psychophysics Toolbox (Brainard, 1997) with a labeled feedback keyboard inside the chamber connected to a testing laptop outside the chamber. Participants could press “Q” (for quit) on the keyboard at any time to immediately end the experiment and be taken out of the chamber. The experimenter sat outside the chamber throughout the entire experiment to speak with or answer any questions from the participant. Participants were also given the option for a break after every task.

#### Stimuli

There were five kinds of stimuli used in this experiment: sounds panning from: (1) front to back; (2) back to front; (3) front, to the middle, and returning to the front; (4) back, to the middle, and returning to the back; and (5) the illusory stimulus (see Figures 2B,C for a visual depiction of each stimulus). The first four are unambiguous in where they begin and end, while the illusory stimulus always remains panned between the two speakers and can be perceived as moving ambiguously in any of the four different trajectories. When hearing these illusory and unambiguous stimuli, a listener has the perception of a sound coming towards them from the distance (either from the front or back), reaching the listener, and then returning to the distance (either toward the front or back).

**Figure 3:**
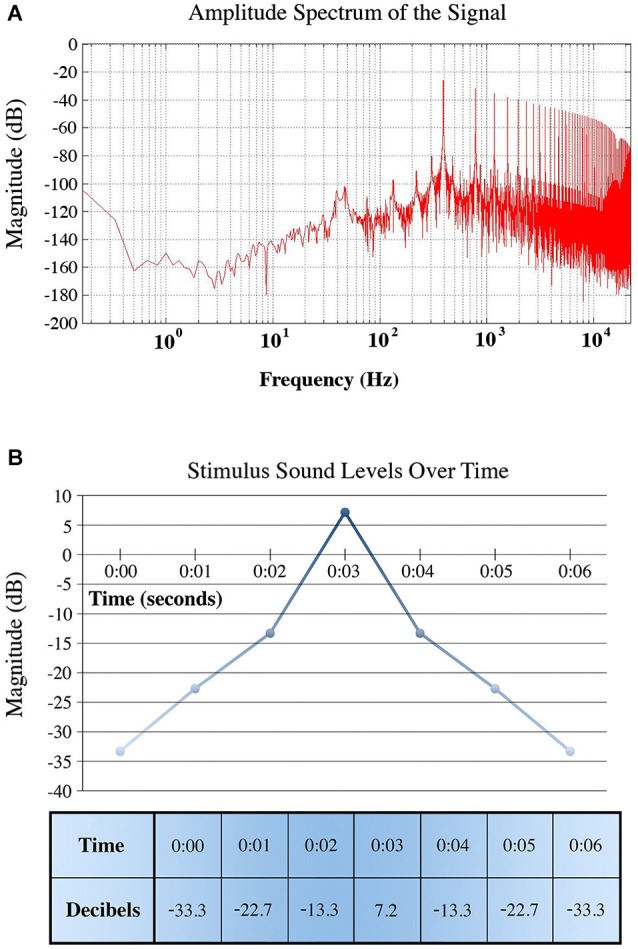
**(A)** Averaged power spectrum for the stimulus. **(B)** A graph and chart depicting the decibel level change used for the stimulus. This increase and decrease in volume is used to model the approach and recession that contributes to the quadri-stability of the illusion. The rate of volume change is intended to depict sound motion traveling at a steady rate.

The base sound for all stimuli was a sawtooth wave with fundamental frequency of 392 Hz, synthesized in Reason (Propellerhead, Stockholm, Sweden) using a Raw_2600_Saw sample in the NN-19 sampler. A power spectrum and plot of volume changes used in our stimulus are shown in Figure 3. The peak volume of the stimulus corresponded to approximately 80 dB SPL (sound pressure level) at the speaker and 65 dB at the listener position. In the case of the unambiguous stimuli, the sound was panned linearly between speakers, and at peak volume was equally balanced at midpoint. In the case of the illusion, the sound is always played at identical intensities in both front and back speakers, with the same change in volume. All panning was done in a quad (four speaker) set-up in Pro Tools (Avid) equipped with the Complete Production Toolkit (Avid). The stimuli were preceded by a preparatory beep, and a pair of beeps an octave apart followed the stimuli to prompt participant feedback.

#### Experimental procedures

##### Part 1: confidence ratings of sound motion perception

In the first task, for each trial the participants heard a pre-recorded vocal cue coming from all speakers indicating the direction of the sound (e.g., “front-back,” meaning a sound that started in the front and traveled to the back), followed either by the illusion or the unambiguous stimulus matching the cue. At this time, none of the participants were aware that there were two types of stimuli (illusory and unambiguous). The participants then rated their confidence in correctly perceiving the motion of the sound on a Likert scale from one to seven. The higher the number selected, the greater confidence the participant indicated in his/her perception of motion along the direction of the vocal cue. Lower numbers suggested a difficulty or an inability to sense the sound move. This task was included to ensure that any inability to identify the illusion or its trajectory in later tasks (Parts 2 and 3) would not due to confusion and random guessing (which would be indicated by low confidence in this task), but to a changing percept of the illusion. Additionally, this task allowed the participant to become accustomed to the experimental setup and stimuli, and allowed for the possible identification of participants who could not perceive sound movement at all (ultimately, no participants were excluded on the basis of this task).

Half of the trials used the illusory stimulus, while the other half used the unambiguous stimuli. Participants rated each condition (the four unambiguous stimuli and the illusion paired with cues for each of the four percepts) twice, resulting in sixteen trials total (eight illusory, eight unambiguous). The vocal cue lasted for two seconds, followed by a jitter of either zero, two, or four seconds rest. The stimulus, including a preparatory beep and six seconds of the actual sound, lasted for a total of seven seconds. After the stimulus, the participants had two seconds to respond with their confidence rating. Following the response, the jitter was counterbalanced with a rest period of five, three, or one second, resulting in a total trial time of 16 s.

##### Part 2: identifying the trajectories of moving sounds

Participants next performed an identification task: in this task, the illusion stimulus or one of the four unambiguous versions was played (without vocal cues) and they chose which of the four motion percepts they heard: front-back, back-front, front-front, or back-back. The participants remained unaware of the existence of the illusion, but a fifth option was given to choose “not sure,” if they were either confused or able to pick up on the illusion and realized the sound has no determined motion percept. This task allowed us to investigate both whether participants were able to correctly perceive unambiguous motion stimuli (if they were not, then perhaps auditory motion perception in general would be found too difficult a task), and whether participants had a directional bias for their perception of the illusion when played in isolation. Participants performed a total of 96 trials presented at random: half of the trials were the illusion (48 trials) and half were the unambiguous stimuli (48 trials, 12 per condition, see Figures 2B,C). Participants were unaware of the distribution of trials amongst conditions. Each trial lasted twelve seconds, with the seven second stimulus, a three second guess period for registering the response, and a two second rest period.

##### Part 3: distinguishing illusory and real sounds

Once this task was complete, the experimenter described to the participant the existence of the illusion and how it worked: they were told that the illusion was a sound that simply increased and decreased in volume in both front and back speakers simultaneously. It was also reiterated that the unambiguous sounds were panned between the speakers through a transfer of volume intensity, while the illusion stayed panned center. The participants then heard the stimuli, once again prompted by the directional vocal cues (front-back, back-front, front-front, back-back), but now chose if each stimulus they heard was “real” (i.e., unambiguous) or “illusion”. With this, we could see if participants could distinguish between the illusion and the unambiguous sounds. This section featured the same sixteen-second trial layout as the first task (See Section Part 1: Confidence ratings of sound motion perception). Participants performed 96 total trials, with 48 trials of the illusion (12 per vocal cue) and 48 trials of the unambiguous stimuli (12 per condition, Figures 2B,C). Trials were again presented in random order.

##### Part 4: rotated control experiment

The experiment concluded with a control task in which the participants were rotated 90° to their left and did a “right-left” trajectory identification task that was essentially identical to the second task (See Section Part 2: Identifying the trajectories of moving sounds). For this final control task, the same sounds continued to come out of the same speakers, but these speakers were now to the right and left of the participant, rather than the front and back (because of the 90° rotation of the participant). The participant heard a sound (either a moving unambiguous sound or the illusion), and responded with the direction in which it was moving (i.e., right-left, left-right, right-right, or left-left), or if it was the illusory sound (with no right-left movement). This control was included to ensure that any effects found in Parts 1–3 were solely due to the nature of the illusory stimulus, as opposed to being due to the methods of the experiment (e.g., the instructions, the testing chamber, or a general inability to perceive moving sound stimuli). This also provided a benchmark of maximum human performance when using unambiguous stimuli. Because the illusion was played at the right and left ears, there was no longer front-back confusion, thus eliminating the multiple percepts of this single stimulus. The timing of the trials was in the twelve-second format of the second task. Participants heard unambiguous stimuli 24 times (six times for each of the four conditions), and the illusion 24 times, for a total of 48 trials.

### Results

#### Part 1: confidence ratings of sound motion perception

Overall confidence ratings were consistently high in the first task. The average rating for the unambiguous sounds was 5.6 out of 7, and the average illusion rating was 5.4, showing no significant difference between the unambiguous and illusion ratings, as well as no difference for the four motion directions, in a 2-factor repeated measures ANOVA (factor 1, unambiguous vs. illusion: *p* > 0.5; factor 2, four motion directions: *p* > 0.1; interaction: *p* > 0.1). Based on the equally high confidence rankings for both conditions, participants seemed to sense the illusion moving just as much as the unambiguous stimuli.

#### Part 2: identifying the trajectories of moving sounds

**Figure 4:**
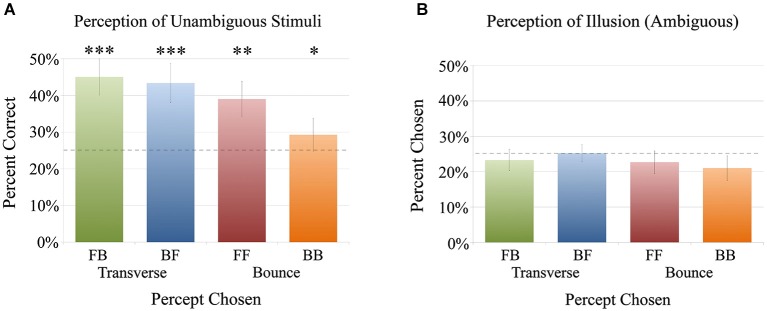
**Results of Experiment 1: (A)** A chart showing the percentage correct of participant’s answers for each of the four unambiguous stimuli. Participants significantly identified the motions of all unambiguous stimuli correctly, and all are above chance level (the dotted line at 25%). The stars indicate number of comparisons that are significant (with *p* < 0.05, see Section Part 2: Identifying the trajectories of moving sounds for specific statistics); both front-back (FB) and back-front (BF) were significantly chosen over all other percepts, while front-front (FF) was only significantly chosen over FB and BB, while back-back (BB) was only significantly chosen over FB. **(B)** A chart showing the percentage of participants’ answers for each direction when hearing the same illusion stimulus. They showed no bias in how they perceived the illusion, with all answers close to chance level (the dotted line at 25%), and no significant differences between answers. Although the option was given to choose “not sure,” participants rarely used it (with an average of only 7.8%).

In this identification task, participants heard either the illusion (ambiguous stimulus) or the unambiguous stimuli, and selected which of the four motion percepts they heard from the transverse conditions of front-back or back-front, and the bounce conditions of front-front, or back-back. Note that participants remained unaware of the existence of the illusion. When guessing the directions of the unambiguous stimuli, the participants had a tendency to correctly identify the trajectories (Figure 4A), with the correct answer being the most chosen trajectory for all unambiguous stimuli. As we expected, people did not respond with 100% accuracy because although they were identifying these unambiguous sounds correctly, the effect of front-back confusion still posed a challenge in perceiving any kind of front or back sound localization. For all unambiguous sounds, participants chose the correct answer most often, based on an ANOVA followed by paired *t*-tests (see Figure 4A). For front-back, they correctly chose front-back significantly over all other options (vs. back-front: *t*_(24)_ = 2.91, *p* < 0.005; vs. front-front: *t*_(24)_ = 4.85, *p* < 10^−4^; vs. back-back: *t*_(24)_ = 6.02, *p* < 10^−5^). Similarly, for back-front they chose back-front significantly over all other options (vs. front-back: *t*_(24)_ = 2.08, *p* < 0.05; vs. front-front: *t*_(24)_ = 4.58, *p* < 0.0005; vs. back-back: *t*_(24)_ = 4.35, *p* < 0.0005). For front-front, while they chose it most often (choosing it 39.0% of the time, compared with a chance level of 25%), the difference was significant in comparison to back-front (*t*_(24)_ = 3.34, *p* < 0.005) and back-back (*t*_(24)_ = 4.44, *p* < 0.0005). Lastly, for back-back, participants again chose back-back most often (29.3% of the time), although the difference was only significant in comparison to front-front (*t*_(24)_ = 2.86, *p* < 0.01).

On the other hand, for the illusion, all possible answers were chosen equally, with no significant difference between them, suggesting no bias towards a specific percept (Figure 4B). Therefore, the participants interpreted the illusion in all of the four possible percepts, with an equal distribution of answers. Although the results were at chance level, participants rarely used the “not sure” key (with an average of 7.8% when hearing the illusion) and gave high confidence ratings in the previous task, suggesting that they truly did perceive the illusion as these four percepts evenly, rather than answering at random.

#### Part 3: distinguishing real and illusory stimuli

**Figure 5:**
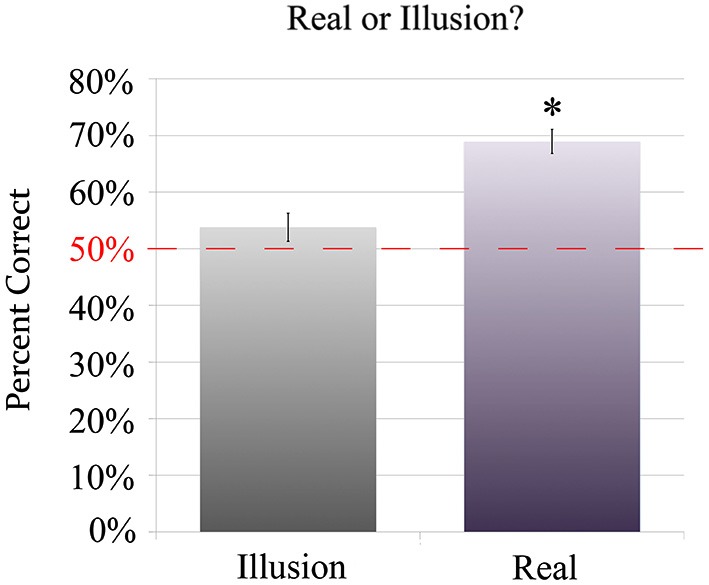
**Results of Experiment 1: A chart showing the percentage of times participants correctly identified a sound as either “illusion” (I) or “real” (R) for sounds that were in fact either the illusion or one of the unambiguous “real” stimuli**. Even when aware of the existence of an illusion stimulus, participants were not able to identify the illusion in the “real or illusion?” task, with no significant difference in identification performance from a chance level of 50% (*M* = 53.92%, SD = 15.90%). However, they were able to identify the unambiguous stimulus as “real” significantly higher than chance (*M* = 68.91%, SD = 15.02%), aligning with their ability to identify the directions of the unambiguous stimuli in the second task of Experiment 1. The star indicates statistical significance between the conditions (*p* < 10^−5^).

When determining if a sound was “real” (unambiguous) or “illusory”, the participants were able to significantly identify that unambiguous stimuli were “real,” with a performance (*M* = 68.91%, SD = 15.02%) significantly greater than a chance level of 50% (*t*_(24)_ = 6.30, *p* < 10^−5^), Figure 5. This paralleled their ability to identify the motions of the unambiguous stimuli (transverse being better identified than bounce) in the second task. However, when faced with the illusion, participants equally identified it as “illusion” or “real”, with no significant difference in their performance from a chance level (*t*_(24)_ = 1.23, *p* > 0.1). This shows that while participants could recognize that a sound was unambiguous when they heard it, they were unable to distinguish the illusion from the unambiguous stimuli (Figure 5).

#### Part 4: rotated control experiment

The final task provided a solid control, using left-right sounds instead of front-back. As expected, the participants had high performance, with a mean performance across subjects of 81.2% correct (vs. a chance level of 20%, *t*_(24)_ = 5.87, *p* < 10^−5^) for all five conditions (the four motion percepts and the illusion), and 69.5% correct for correctly identifying the illusion, showing that participants could correctly complete the task and distinguish the illusion from the ambiguous sounds when the effect was removed, as this is a phenomenon exclusive to the front-back domain.

## Experiment 2—testing perceptual biases of the illusion

Since the effectiveness of the illusion has been established in Experiment 1, the next question was to what degree this illusion produced the phenomena of other multi-stable illusions, such as perceptual switching (Orlandi, 2012), locking (Kayahara, 2003), and biases (Troje and McAdam, 2010). Many multi-stable illusions experience perceptual switching, where the observer’s perception of the illusion switches (either spontaneously or intentionally) amongst the possible options (Iig et al., 2008; Orlandi, 2012). People also experience a perceptual locking where a participant gets locked in one percept of an illusion over a period of time, such as with the Spinning Dancer illusion (Kayahara, 2003) or with visual hybrids (Brady and Oliva, 2012). Additionally, observers of multi-stable illusions often have a bias for which percept is more likely to be perceived first or over an extended period of time (Troje and McAdam, 2010).

For Experiment 2, using headphones, we quantified how much perceptual switching, locking, and biasing occur in the quadri-stable illusion. We know from other works that front-back reversal occurs with different vocal stimuli presented in headphones (spoken words, Gilkey and Anderson, 1995). For instance, Begault and Wenzel (1993) found that the degree of reversals for speech stimuli is asymmetrical between front-back and back-front (with reversals from front to back higher than back to front, see also Wightman and Kistler, 1989). Additionally, multimodal illusion work has shown that visual cues can affect the auditory perception of an illusion and vice versa (McGurk and MacDonald, 1976; Sekuler et al., 1997). To ensure that participants did not use visual cues to influence perception of auditory motion, we conducted this experiment with two sets of participants—one set passively viewing a blank screen, and another set blindfolded—and compared their results.

### Materials and methods

#### Participants

Twenty-six participants (11 female) with self-reported normal hearing between the ages of 18–35 participated in the study. Sixteen participants passively viewed a computer screen during the experiment (“sighted condition”), while ten participants were blindfolded for the duration of the study (“blindfolded condition”). All participants consented to the experiment following guidelines set by the MIT Institutional Review Board. Prior to beginning the experiment, each participant’s ability to hear in the frequency of the stimulus in each ear was tested using part of a pure tone audiometry in the same manner as the first experiment (Wolfe et al., 2005). Participants were compensated 10 for the 30 min experiment.

#### Testing apparatus

The experiment was designed and run in PsychoPy (The University of Nottingham). All participants were seated in a quiet window-less room at a computer and used the same pair of Sony MDR-NC7 noise canceling headphones to perform the experiment. Participants viewed experimental instructions on the computer, which were also explained verbally by the experimenter. Blindfolds were not put on until the instructions were read and understood. Responses were made on the keyboard.

#### Stimuli

The audio stimulus was the same as that used in Experiment 1, but now began and ended at the peak volume (perceptually centered at the listener) and was played in headphones at 60 dB SPL. The sound was looped continuously for this experiment; practically, the sound’s volume decreased and increased in waves spanning six seconds from peak to peak, lasting for 1 min total (resulting in ten cycles). The sound remained panned center (between left and right) for its entire duration. A preparatory beep alerted participants to the beginning of each stimulus.

#### Experimental procedures

Sighted and blindfolded participants participated in the same set of experimental procedures. The illusion stimulus was first explained. The participants were told that the sound could be interpreted as moving to the front or back, and that the sound was in the middle of its trajectory (at the observer) when it reached its peak in volume. The participants were informed that sometimes the sound might bounce off of them or travel through them to the opposite side, resulting in the four different percepts of the illusion. Additionally, whenever the sound reached its lowest volume, it could seem to spontaneously relocate to the opposite side before returning to the listener. The participants listened to a demonstration of the stimulus sound played continuously for 1 min to see if they were able to perceive this front and back motion.

In the main experiment, participants heard the illusion playing continuously for 1 min, and pressed keys through the duration of the stimulus to indicate if the sound was in front, in back, or in the middle (when the sound reached the participant). During this one minute, the sound made ten loops (from the middle, outwards, and then back to the middle), and adjacent loops were conceptually paired for the analysis (into percepts of front-back, back-front, front-front, and back-back, as in Experiment 1). Key presses were counterbalanced—half of the participants used the left arrow key to indicate “front,” and the right to indicate “back,” while the other half of participants had these keys reversed, with the right arrow key being “front.” In all cases, the down arrow key was used to indicate “middle” or “at the listener.” We avoided using the up and down keys for front and back to minimize any influence of motor response on the perception of the illusion. After the sound was played, a ten second break was provided to refresh any perceptual locking. After seven iterations of the 1 min stimulus, participants received an untimed break and continued with a keypress. Overall, the participants performed 21 runs of 1 min.

### Results

**Figure 6:**
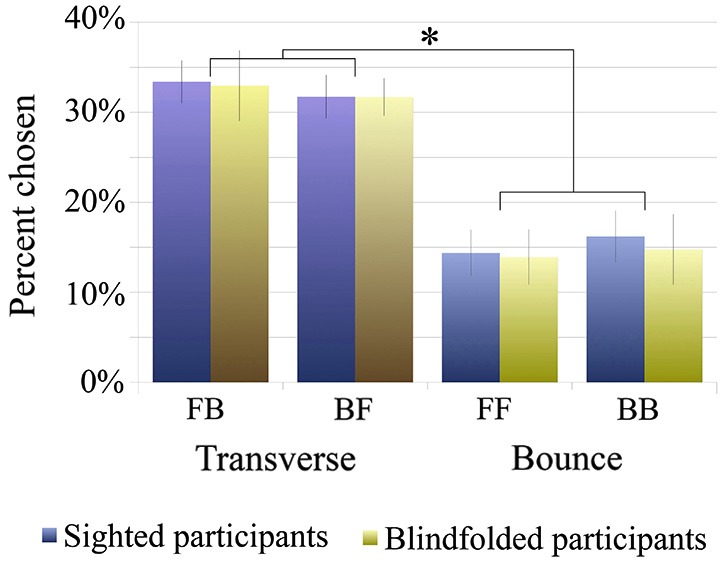
**Results of Experiment 2: Percent chosen of responses for each of the four conditions, front-back (FB), back-front (BF), front-front (FF) and back-back (BB) for sighted (blue) and blindfolded (orange) groups**. When the illusion was heard continuously, participants had a significant transverse (front-to-back and back-to-front) bias over the bounce (front-to-front and back-to-back) percepts (the star indicates a significant effect in a 2-factor ANOVA, *p* < 10^−9^). This bias was identical between the sighted and blindfolded participant groups.

Based on a 2-factor between-subjects ANOVA, there were no significant differences in how sighted vs. blindfolded participants perceived the illusion (*p* > 0.5). However, there was a significant difference in transverse conditions being perceived significantly more than the bounce conditions across both participant groups (*F*_(1,48)_ = 61.77, *p* < 10^−9^; See Figure 6). These results point to a bias for transverse (front-back or back-front) percepts over bounce (front-front or back-back) percepts, and also provide evidence that one’s percept is not affected by whether the eyes are opened or covered. The significantly higher percepts of transverse conditions vs. bounce were present within each participant group as well (paired-sample *t*-tests, sighted: *t*_(15)_ = 4.20, *p* < 0.001; blindfolded: *t*_(9)_ = 4.20, *p* < 0.005). However, there was no significant difference within the transverse condition (sighted: *t*_(15)_ = 0.94, *p* > 0.1, blindfolded: *t*_(9)_ = 0.31, *p* > 0.5), nor within the bounce condition (sighted: *t*_(15)_ = 0.55, *p* > 0.5, blindfolded: *t*_(9)_ = 0.17, *p* > 0.5), showing no specific bias beyond a tendency to choose transverse conditions.

**Figure 7:**
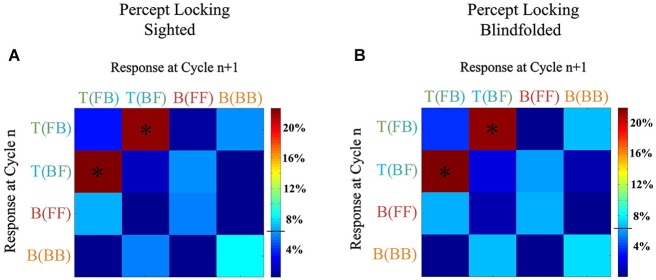
**Results of Experiment 2: Transition matrices between the response chosen at cycle (trial) *n*, and at the next cycle (trial *n* + 1) for the sighted group (A) and blindfolded group (B)**. Participants became significantly locked alternating between the two transverse percepts (front-to-back and back-to-front), as indicated by the stars on the red cells in this graph (*p* < 0.005). The y-axis shows the initial percept (percept at cycle *n*), and the *x*-axis shows which percept follows (percept at cycle *n* + 1). Chance is at 6.25%, indicated by the horizontal line in the color-scale to the right. The two groups exhibited the same transition matrices.

Figure 7 illustrates the percentage of time people switched from one interpretation to another one (chance level being 6.25%): if the transition between all of four percepts was the same, the probability matrix would be homogenous (in Figure 7, all would be blue cells, at chance level). When analyzing which percepts followed which, a continuity bias can be seen in the probability matrices of becoming locked in alternating transverse conditions; front-back percepts are followed by back-front percepts and, similarly, back-front percepts are followed by front-back percepts, significantly more than all other percepts (all *p* < 0.005). In contrast, participants did not significantly get locked in bouncing conditions; no pair of percepts was perceived significantly higher than all others. Additionally, the two groups (sighted and blindfolded participants) behaved the same (correlation of the transition matrices of sighted and blindfolded: *r* = 0.998, *p* ~ 0.00).

## Discussion

Here, we present a novel quadri-stable auditory illusion that uses aspects of front-back confusion and the growing-louder effect (Reinhardt-Rutland and Ehrenstein, 1996) to create an ambiguous dynamic sound that observers can perceive as moving along four different possible trajectories. This auditory localization illusion joins the tradition of multi-stable illusions in the visual domain (e.g., the Ames, 1951; de Heer and Papathomas, in press) and in auditory pitch (e.g., Shepard tone, Shepard, 1964; Deutsch, 1992).

Our first experiment demonstrates that the illusion can flexibly produce four different motion percepts, and is almost indistinguishable from unambiguously moving stimuli. Participants are equally confident in perceiving the motion of all stimuli (both the unambiguous stimuli and the illusion). Additionally, the participants are able to correctly identify the trajectories along which the unambiguous stimuli are moving, and guess that these stimuli are “real” rather than illusory. This indicates that participants can accurately identify auditory motion when it is unambiguous. However, when listening to the ambiguous illusion, participants divide their interpretations of the illusion equally between the four unambiguous percepts, showing no strong bias for any direction when the illusion is played only a single time. Their high confidence ratings for perceiving motion in the illusory stimulus suggest this lack of bias should not be the result of choosing percepts at random. Neglecting to choose the “not sure” key during the identification task parallels their confidence in their perceived motions of the illusion. Participants also perform at chance level when classifying ambiguous stimuli as “real” or “illusory.” These results demonstrate the validity of this quadri-stable illusion, showing that it effectively emulates four different trajectories and is not distinguished from the unambiguous stimuli.

The second experiment examines various characteristics of this illusion, including perceptual locking, switching, and biases. When the illusion is played continuously, participants show a transverse bias—getting locked between percepts going from front to back and back to front. This transverse bias may reflect real-world experience, where some external force must interact with a moving object in order for it to change trajectory (Spelke et al., 1995). In the absence of a physical or visual influence, a continuous trajectory could be the simpler, default percept. Additionally, this experiment shows that the transverse bias is the same regardless of whether or not vision is restricted with a blindfold, showing that this transverse bias is not due to an external visual confound.

At distances close to the observer, ILDs and ITDs are especially important to sound localization, providing distance as well as azimuth information. The near-field ambiguous region where a given set of interaural cues is constant has been described as a “torus of confusion” and is most pronounced at azimuths away from the median plane, degenerating to encompass the entire median plane as source position approaches the midline (Shinn-Cunningham et al., 2000). The illusion only varies overall intensity, with ILD and ITD always at 0, potentially contributing to its ambiguity by eliminating not just lateralization, but the ILD distance cue as well. Future studies could examine the strength and/or frequency dependance of the illusory effect at greater distances and different positions off the median plane via the introduction of ILDs and ITDs. Using different spectral qualities as well as different percept selection options could also add elevation percepts, expanding the illusion from quadri-stable to n-stable.

Visual influences on perception could constitute an important next step in investigating the illusion and the multimodality of object localization in general. The perceptual flexibility of the illusion makes room for exploring ways to alter or bias the interpretation of space. A multimodal study could explore the influence of adding visual stimuli or an additional auditory stimulus on the current transverse bias present when the illusion is played continuously. For example, a distractor flash or tone at the peak volume of the illusion could potentially reduce the transverse bias, similar to the auditory modulation of the visual bounce-stream illusion (Sekuler et al., 1997). This flash or tone could suggest an additional action occurring, such as hitting the listener, causing a change in trajectory (i.e., bouncing). Alternatively, adding dynamic visual stimuli may also alter participant interpretations. For example, a virtual reality interface similar to that used to create the out-of-body illusion (Ehrsson, 2007) depicting a visual object moving along different trajectories could bias perceptions of the illusion. In the real world, vision relies on front-oriented scenery to effectively navigate and avoid obstacles, while the auditory system provides valuable information outside the visual field (e.g., behind the listener). When viewing a dynamic world, it may be harder to perceive an imaginary, and therefore invisible, object as being in front of the listener (thus influencing a bias for sounds behind the listener). As a result, the Transverse-and-Bounce Auditory Illusion could potentially be interpreted differently in early-blind or blind-from-birth listeners, who have not had vision to rely on when interpreting space in front of them. Finally, the illusion could provide insight for several questions in neuroscience, such as the neural mechanisms of auditory spatial localization, multimodal interactions, and perceptual switching.

## Conflict of interest statement

The authors declare that the research was conducted in the absence of any commercial or financial relationships that could be construed as a potential conflict of interest.
